# Identification of circRNA–miRNA–Immune-Related mRNA Regulatory Network in Gastric Cancer

**DOI:** 10.3389/fonc.2022.816884

**Published:** 2022-02-24

**Authors:** Zhenhai Wu, Pengyuan Liu, Ganlu Zhang

**Affiliations:** ^1^ Department of Oncology, Zhejiang Hospital, Hangzhou, China; ^2^ The Second School of Clinical Medicine, Zhejiang Chinese Medical University, Hangzhou, China

**Keywords:** gastric cancer, circRNA, miRNAs, mRNAs, diagnosis, immune, signaling pathway, protein–protein interaction

## Abstract

The pathogenesis of gastric cancer (GC) is still not fully understood. We aimed to find the potential regulatory network for ceRNA (circRNA–miRNA–immune-related mRNA) to uncover the pathological molecular mechanisms of GC. The expression profiles of circRNA, miRNA, and mRNA in gastric tissue from GC patients were downloaded from the Gene Expression Omnibus (GEO) datasets. Differentially expressed circRNAs, miRNAs, and immune-related mRNAs were filtered, followed by the construction of the ceRNA (circRNA–miRNA–immune-related mRNA) network. Functional annotation and protein–protein interaction (PPI) analysis of immune-related mRNAs in the network were performed. Expression validation of circRNAs and immune-related mRNAs was performed in the new GEO and TCGA datasets and *in-vitro* experiment. A total of 144 differentially expressed circRNAs, 216 differentially expressed miRNAs, and 2,392 differentially expressed mRNAs were identified in GC. Some regulatory pairs of circRNA–miRNA–immune-related mRNA were obtained, including hsa_circ_0050102–hsa-miR-4537–NRAS–Tgd cells, hsa_circ_0001013–hsa-miR-485-3p–MAP2K1–Tgd cells, hsa_circ_0003763–hsa-miR-145-5p–FGF10–StromaScore, hsa_circ_0001789–hsa-miR-1269b–MET–adipocytes, hsa_circ_0040573–hsa-miR-3686–RAC1–Tgd cells, and hsa_circ_0006089–hsa-miR-5584-3p–LYN–neurons. Interestingly, FGF10, MET, NRAS, RAC1, MAP2K1, and LYN had potential diagnostic value for GC patients. In the KEGG analysis, some signaling pathways were identified, such as Rap1 and Ras signaling pathways (involved NRAS and FGF10), Fc gamma R-mediated phagocytosis and cAMP signaling pathway (involved RAC1), proteoglycans in cancer (involved MET), T-cell receptor signaling pathway (involved MAP2K1), and chemokine signaling pathway (involved LYN). The expression validation of hsa_circ_0003763, hsa_circ_0004928, hsa_circ_0040573, FGF10, MET, NRAS, RAC1, MAP2K1, and LYN was consistent with the integrated analysis. In conclusion, the identified ceRNA (circRNA–miRNA–immune-related mRNA) regulatory network may be associated with the development of GC.

## Introduction

Gastric cancer (GC) is one of the most serious malignant tumors (1). According to the GLOBOCAN Estimates of Incidence and Mortality Worldwide for 36 Cancers in 185 Countries, the incidence of GC is 7.7% (2). In addition, with 2.26 million new cases estimated in 2020, GC (0.77 million) has become the most commonly diagnosed cancer worldwide, ranking only second to female breast cancer (3). Recurrence is the main cause of GC-related death. Although mortality is steadily decreasing, GC still leads to a poor diagnosis and prognosis for patients (4). In addition, the 5-year survival rate of patients is still very low in serious GC patients. It has been shown that obesity, active tobacco smoking, high meat and salt intake, low vegetable/fruit intake, *Helicobacter pylori* infection, and gut microbiota have been shown to be associated with an increased risk of GC (5–8). In addition, epigenetic alterations are associated with the processes of gastric carcinogenesis and metastasis (9). Clinically, surgery is the only curative treatment; however, some patients have inoperable disease at diagnosis (10). Hence, there is a need to elucidate the potential molecular mechanisms in the development of GC and to look for new molecular markers and therapeutic targets.

circRNAs usually result from splicing/back-splicing events *via* exon or intron circularization (11). circRNA can act as a molecular sponge of miRNA to regulate mRNA expression. It was reported that sponge circRNAs are part of a complex RNA-binding protein–circRNA–miRNA–mRNA interaction network and are involved in the establishment, chemoresistance, and progression of GC (12). hsa_circ_LARP4 can inhibit cell invasion of GC by sponging hsa-miR-424-5p and regulating large tumor suppressor kinase 1 (LATS1) expression (13). hsa_circ_NRIP1 can act as an hsa-miR-149-5p sponge to promote GC progression *via* the AKT serine/threonine kinase 1 (AKT1)/mechanistic target of rapamycin kinase (mTOR) pathway (14). hsa_circ_NHSL1 can promote GC progression through the hsa-miR-1306-3p/SIX homeobox 1 (SIX1)/vimentin axis (15). hsa_circ_CACTIN can promote GC progression by sponging hsa-miR-331-3p and regulating transforming growth factor beta receptor 1 (TGFBR1) expression (16). hsa_circ_0026359 can enhance cisplatin resistance in GC *via* targeting the hsa-miR-1200/DNA polymerase delta 4, accessory subunit (POLD4) pathway (17). In addition, it is believed that the pathogenies and progression of GC are influenced by the cross-talk between tumor cells and the host immune system (18–20). In view of this, we tried to find the potential differentially expressed circRNAs, miRNAs, and immune-related mRNAs in GC.

## Materials and Methods

### Data Retrieval and Analysis

In this study, the expression profiles of circRNA, miRNA, and mRNA were downloaded from the Gene Expression Omnibus (GEO) datasets by searching keywords [“gastric cancer” (All Fields) AND “Homo sapiens”(porgn) AND “gse”(Filter)]. The following datasets were selected: 1) dataset must be genome-wide transcriptome data of mRNA/miRNA/circRNA; 2) data were obtained from tumor tissues of the GC group and paracancer control group; and 3) both standardized and raw datasets were considered. Finally, two circRNA expression datasets (GSE83521 and GSE89143), two miRNA expression datasets (GSE93415 and GSE158315), and two mRNA expression datasets (GSE66229 and GSE65801) were selected (**Table 1**).

**Table 1:** Detailed information of selected datasets of circRNA, miRNA, and mRNA expression datasets.

	GEO accession	Author	Platform	Samples (N:GC)	Year	Tissue
circRNA	GSE83521	Yan Zhang	GPL19978 Agilent-069978 Arraystar Human CircRNA microarray V1	6:6	2017	Gastric tissue
	GSE89143	Junming Guo	GPL19978 Agilent-069978 Arraystar Human CircRNA microarray V1	3:3	2017	Gastric tissue
miRNA	GSE93415	Marek Sierżęga	GPL19071 Exiqon miRCURY LNA microRNA array; 7th generation REV - hsa, mmu, and rno; batch 208520-22; lot 35101-35101 (miRBase 19.0)	20:20	2017	Gastric tissue
	GSE158315	Yuping Wang	GPL18058 Exiqon miRCURY LNA microRNA array, 7th generation (miRBase v18, condensed Probe_ID version)	5:5	2021	Gastric tissue
mRNA	GSE66229	Michael Nebozhyn	GPL570 (HG-U133_Plus_2) Affymetrix Human Genome U133 Plus 2.0 Array	100:300	2015	Gastric tissue
	GSE65801	Hao Li	GPL14550 Agilent-028004 SurePrint G3 Human GE 8 × 60K Microarray (Probe Name Version)	32:32	2015	Gastric tissue

N, paracancer control group; GC, gastric cancer group.

### Screening of Differentially Expressed circRNAs, miRNAs, and Immune-Related mRNAs

The probe and ID of circRNA/miRNA/mRNA were mapped one by one. After scale standardization, the dataset was merged and batch effects were removed *via* the ComBat function of sva package (R-4.0.5). The metaMA and limma packages were used to identify circRNAs/miRNAs/mRNAs. The default parameter of the Pvalcombination command of the metaMA package was used to make the difference. *p*-values and effect sizes (ES, the effectSize obtained from the metaMA package) from data were calculated either from classical or moderated *t*-tests. These *p*-values were combined by the inverse normal method. The Benjamini–Hochberg threshold was used to calculate the false discovery rate (FDR). |Combined.ES| >1 and FDR <0.05 were the screening criteria for circRNAs/miRNAs/mRNAs. In addition, immune-related mRNAs were downloaded from the ImmPort database (https://www.immport.org/shared/home). Those immune-related mRNAs were obtained by intersection of differentially expressed mRNAs and immune-related mRNAs in the ImmPort database. xCell (21) was used to calculate the distribution of immune cells in each sample based on the ssGSEA method. xCell score was organized into immune cell infiltration matrix to calculate the types of immune cells that differed between the GC group and the normal control group. The Pearson correlation coefficient method was used to calculate the correlation between mRNAs and differential immune cells.

### Construction of the ceRNA (circRNA–miRNA–Immune-Related mRNA) Regulatory Network

The TargetScan (http://www.targetscan.org/vert_71/) software was utilized to predict targeted relationship between differentially expressed circRNAs and differentially expressed miRNAs. In addition, the miRWalk software (http://mirwalk.umm.uni-heidelberg.de/interactions/) was applied to predict targeted relationship between miRNAs and mRNAs. The relationship pairs of miRNA–mRNA verified in at least one database (TargetScan, miRDB, and MiRTarBase) were selected. The overlapping mRNAs were obtained between predicted mRNAs (in the miRNA–mRNA relationship pairs) and immune-related differential expression mRNAs. The ceRNA (circRNA–miRNA–immune-related mRNA) regulatory network was constructed by fusing with the circRNA–miRNA relationship pairs and miRNA–immune-related mRNA relationship pairs.

### Functional Annotation of Immune-Related mRNAs in the ceRNA Regulatory Network

To investigate the function of immune-related mRNAs in the regulatory ceRNA network, Gene Ontology (GO) and Kyoto Encyclopedia of Genes and Genomes (KEGG) analyses were performed by using the DAVID database (https://david.ncifcrf.gov/tools.jsp). FDR <0.05 was considered as statistical significance.

### Protein–Protein Interaction Network of Immune-Related mRNAs in the ceRNA Regulatory Network

To further explore the interaction between immune-related mRNAs in the ceRNA regulatory network, protein–protein interaction (PPI) was performed by using the STRING database. The results were imported using the Cytoscape software (http://www.cytoscape.org/). The CytoHubba plug-in was used to filter core immune-related mRNAs by intersecting the first 10 mRNAs of each algorithm (degree, MNC, MCC, and EPC). In addition, the ROC analysis was carried out to assess the diagnostic value of core immune-related mRNAs in the PPI network.

### Expression Validation of Differentially Expressed circRNAs and Immune-Related mRNAs

In order to further validate the expression of identified circRNAs and immune-related mRNAs, electronic validation was performed. The GSE93541 dataset (involving tumor tissues from three cases and three normal controls) and the GSE141977 dataset (involving plasma from three cases and three normal controls) were used for expression validation of identified circRNAs. Additionally, the Cancer Genome Atlas (TCGA) dataset (involved tumor tissues from 375 cases and 32 normal controls) was used to validate the expression of identified mRNAs. The expression result of these circRNAs and mRNAs was shown by the box plots. Statistical significance was ascribed to *p*-value <0.05.

In addition, qRT-PCR was used to further validate the expression of identified circRNAs and immune-related mRNAs. Five GC patients and five normal controls were enrolled. The blood sample of these individuals was collected. All participating individuals provided informed consent with the approval of the Ethics Committee of Zhejiang Hospital (2021-164K). Total RNA of the blood sample was extracted and DNA was synthesized using FastQuant cDNA first-strand synthesis kit. qRT-PCR was performed in the SuperReal PreMix Plus (SYBR Green). Relative circRNA/mRNA expression was analyzed by the 2^−△△CT^ method. 2^−△△CT ^>1 and 2^−△△CT ^<1 represented upregulation and downregulation, respectively.

## Results

### Differentially Expressed circRNAs, miRNAs, and Immune-Related mRNAs

**Figure 1:**
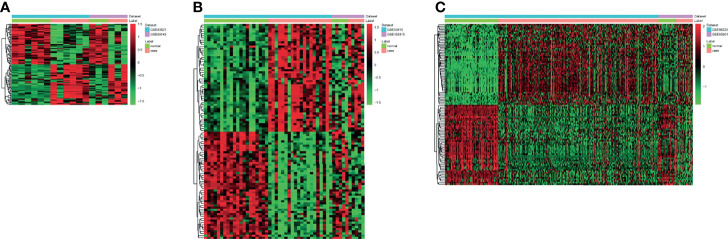
The heat map of the top 100 differentially expressed circRNAs **(A)**, miRNAs **(B)**, and mRNAs **(C)** in gastric cancer (GC).

**Figure 2:**
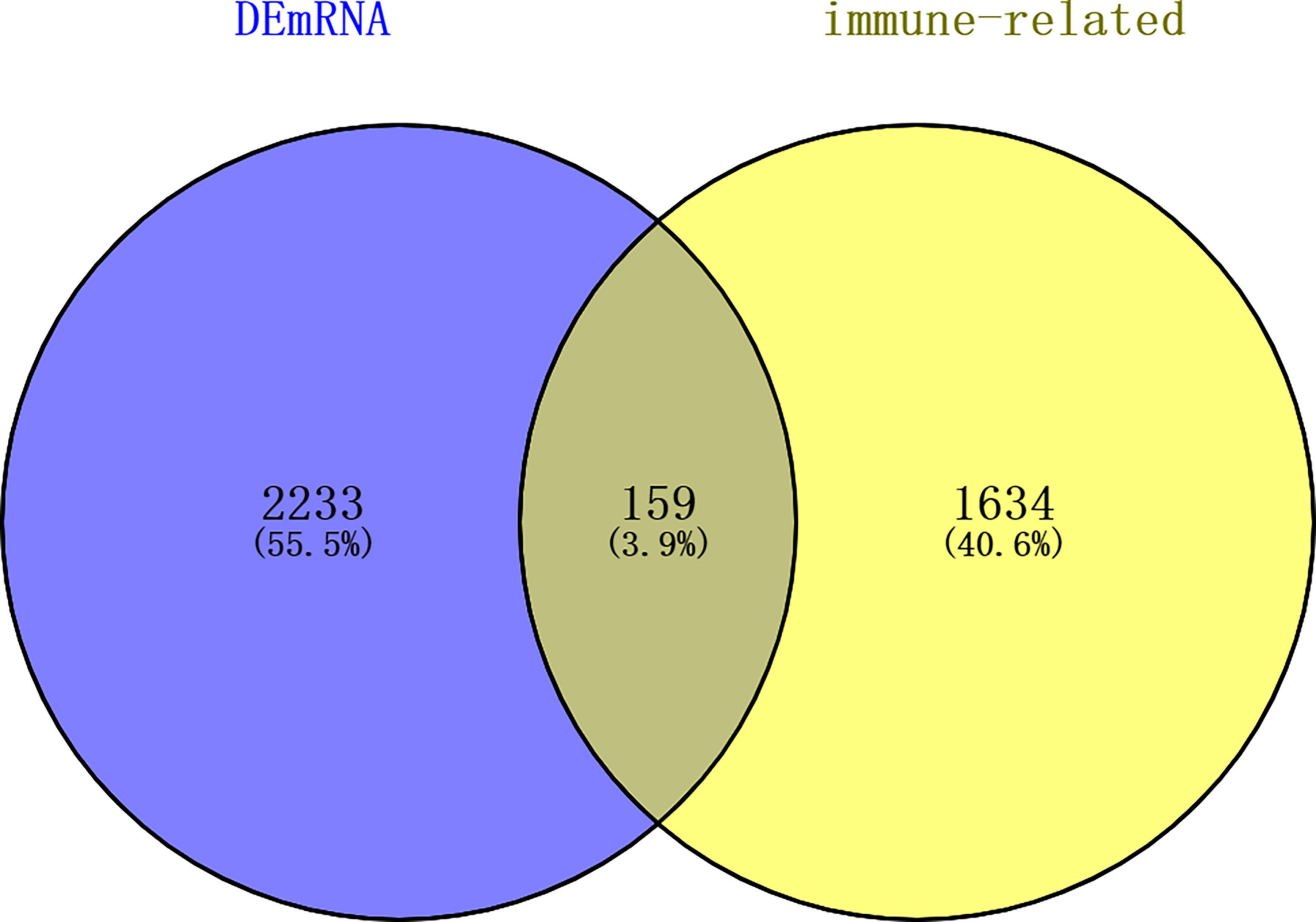
Venn diagram of differentially expressed mRNAs in GC and immune-related mRNAs in the ImmPort database. dEmRNA, differentially expressed mRNA.

A total of 144 differentially expressed (55 upregulated and 89 downregulated) circRNAs, 216 differentially expressed (105 upregulated and 111 downregulated) miRNAs, and 2,392 differentially expressed (1,329 upregulated and 1,063 downregulated) mRNAs were identified in GC. The top 10 differentially expressed circRNAs were identified, such as hsa_circ_0045602 (upregulation), hsa_circ_0006089 (upregulation), hsa_circ_0001789 (upregulation), hsa_circ_0018004 (downregulation), and hsa_circ_0003763 (downregulation) (**Table 2**). hsa-miR-4537 was one of top 10 downregulated miRNAs (**Table 3**). The top 10 differentially expressed mRNAs are listed in **Table 4**. The heat map of the top 100 differentially expressed circRNAs, miRNAs, and mRNAs is shown in **Figures 1A–C**, respectively. In addition, 1,793 immune-related mRNAs were obtained from the ImmPort database. It was noted that 159 immune-related mRNAs were also differentially expressed mRNAs in GC (**Figure 2**).

**Table 2:** The top 10 upregulated and downregulated circRNAs in GC.

ID	Symbol	Combined.ES	*p*-value	FDR	Up/down
hsa_circRNA_102191	hsa_circ_0045602	3.118562462	1.07E−06	0.000830662	Up
hsa_circRNA_104947	hsa_circ_0007613	3.076836769	1.43E−06	0.000830662	Up
hsa_circRNA_101882	hsa_circ_0040573	3.859880212	1.82E−06	0.000830662	Up
hsa_circRNA_102592	hsa_circ_0052001	3.180131272	2.28E−06	0.000830662	Up
hsa_circRNA_101471	hsa_circ_0034398	2.865182594	3.78E−06	0.000836756	Up
hsa_circRNA_102614	hsa_circ_0006089	2.831849087	3.94E−06	0.000836756	Up
hsa_circRNA_104589	hsa_circ_0001789	3.306599675	4.20E−06	0.000836756	Up
hsa_circRNA_100641	hsa_circ_0019054	2.89673611	4.77E−06	0.00087084	Up
hsa_circRNA_101875	hsa_circ_0040481	2.642927097	9.51E−06	0.001488245	Up
hsa_circRNA_102777	hsa_circ_0055521	2.42548772	2.78E−05	0.003039928	Up
hsa_circRNA_100571	hsa_circ_0018004	−3.196809378	9.51E−07	0.000830662	Down
hsa_circRNA_104599	hsa_circ_0001793	−3.624031186	2.17E−06	0.000830662	Down
hsa_circRNA_400066	hsa_circ_0092330	−2.876804101	3.17E−06	0.000836756	Down
hsa_circRNA_101651	hsa_circ_0036941	−2.829679697	3.88E−06	0.000836756	Down
hsa_circRNA_001914	hsa_circ_0000902	−2.987023494	7.15E−06	0.001204509	Down
hsa_circRNA_101965	hsa_circ_0000740	−2.632340492	1.05E−05	0.001531677	Down
hsa_circRNA_103442	hsa_circ_0003763	−2.780691514	1.88E−05	0.00256875	Down
hsa_circRNA_400056	hsa_circ_0092297	−2.597537412	2.21E−05	0.00275069	Down
hsa_circRNA_100382	hsa_circ_0007277	−2.696712581	2.26E−05	0.00275069	Down
hsa_circRNA_104661	hsa_circ_0004366	−2.495771422	2.39E−05	0.00275519	Down

ES, effect size; FDR, false discovery rate.

**Table 3:** The top 10 upregulated and downregulated miRNAs in GC.

Symbol	Combined.ES	*p*-value	FDR	Up/down
hsa-miR-181a-5p	2.621880675	8.01E−13	6.84E−10	Up
hsa-miR-181b-5p	2.541945488	4.05E−12	1.15E−09	Up
hsa-miR-23a-3p	2.283816919	1.17E−10	1.11E−08	Up
hsa-miR-331-3p	2.121044968	1.04E−09	6.82E−08	Up
hsa-miR-320c	2.029389583	1.53E−09	8.73E−08	Up
hsa-miR-25-3p	1.94259155	4.78E−09	2.55E−07	Up
hsa-miR-320d	1.961475455	6.29E−09	2.98E−07	Up
hsa-miR-92a-3p	1.928060578	7.06E−09	3.02E−07	Up
hsa-miR-21-3p	1.858793042	1.58E−08	5.18E−07	Up
hsa-miR-320e	1.904827788	1.72E−08	5.44E−07	Up
hsa-miR-4279	−2.564714404	1.76E−12	7.51E−10	Down
hsa-miR-3124-3p	−2.474608778	5.60E−12	1.19E−09	Down
hsa-miR-4728-3p	−2.455455298	1.21E−11	2.07E−09	Down
hsa-miR-4635	−2.393092709	1.54E−11	2.19E−09	Down
hsa-miR-4537	−2.418295745	5.96E−11	7.27E−09	Down
hsa-miR-642b-5p	−2.298345262	8.63E−11	9.21E−09	Down
hsa-miR-5196-3p	−2.172908344	3.75E−10	3.20E−08	Down
hsa-miR-877-3p	−2.202854847	4.82E−10	3.51E−08	Down
hsa-miR-4290	−2.27123672	4.93E−10	3.51E−08	Down
hsa-miR-4268	−2.219462893	1.40E−09	8.54E−08	Down

ES, effect size; FDR, false discovery rate.

**Table 4:** The top 10 upregulated and downregulated mRNAs in GC.

ID	Symbol	Combined.ES	*p*-value	FDR	Up/down
9997	SCO2	1.400471	<0.001	<0.001	Up
9991	ROD1	1.325606	<0.001	<0.001	Up
9966	TNFSF15	1.160469	<0.001	<0.001	Up
995	CDC25C	1.639835	<0.001	<0.001	Up
994	CDC25B	1.62622	<0.001	<0.001	Up
993	CDC25A	1.407589	<0.001	<0.001	Up
9928	KIF14	2.287231	<0.001	<0.001	Up
9926	LPGAT1	1.19746	<0.001	<0.001	Up
9918	NCAPD2	1.645622	<0.001	<0.001	Up
991	CDC20	1.774075	<0.001	<0.001	Up
9992	KCNE2	−1.72329	<0.001	<0.001	Down
9934	P2RY14	−1.90515	<0.001	<0.001	Down
9905	SGSM2	−1.00543	<0.001	<0.001	Down
9892	SNAP91	−1.27282	<0.001	<0.001	Down
9886	RHOBTB1	−1.07693	<0.001	<0.001	Down
9874	TLK1	−1.11589	<0.001	<0.001	Down
9867	PJA2	−1.7726	<0.001	<0.001	Down
9832	JAKMIP2	−1.09529	<0.001	<0.001	Down
9829	DNAJC6	−1.03815	<0.001	<0.001	Down
9783	RIMS3	−1.64884	<0.001	<0.001	Down

ES, effect size; FDR, false discovery rate.

### ceRNA (circRNA–miRNA–Immune-Related mRNA) Regulatory Network

**Figure 3:**
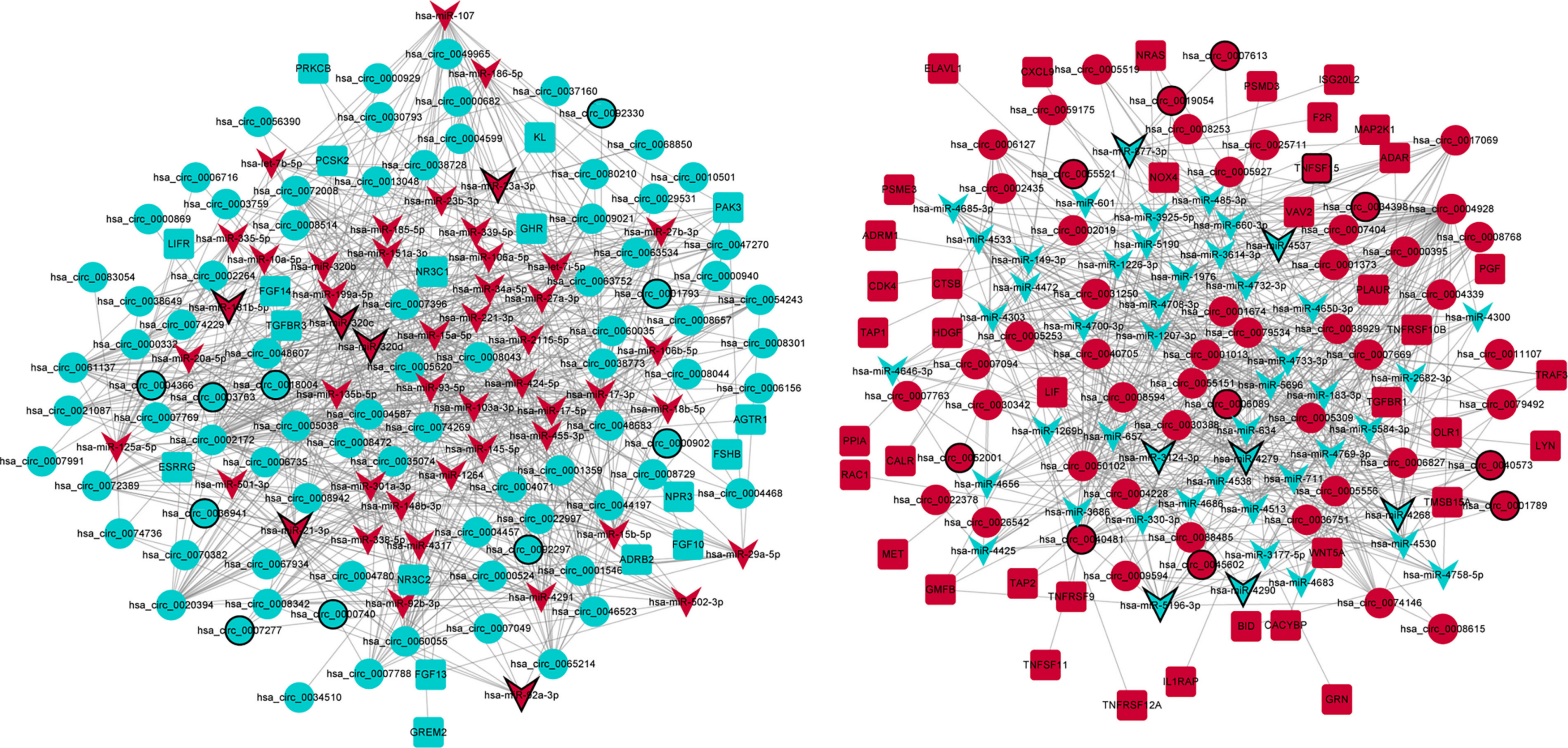
The ceRNA (circRNA–miRNA–immune-related mRNA) regulatory network in GC. Circle, V shape, and rectangle represent differentially expressed circRNA, miRNA, and mRNA, respectively. Red and green represent upregulation and downregulation, respectively. The black border represents the top 10 up/downregulated circRNAs/miRNAs/mRNAs.

A total of 2,037 negatively regulated targeting relationship pairs of circRNA–miRNA (involving 143 circRNAs and 198 miRNAs) and 142 negatively regulated targeting relationship pairs of miRNA–immune-related mRNA (involving 97 miRNAs and 58 immune-related mRNAs) were identified *via* the TargetScan software and miRWalk, respectively. The ceRNA (circRNA–miRNA–immune-related mRNA) regulatory network (involving 137 circRNAs, 96 miRNAs, and 58 immune-related mRNAs) was obtained by fusing with the circRNA–miRNA relationship pairs and miRNA–immune-related mRNA relationship pairs (**Figure 3**). Some ceRNA relationship pairs were identified, such as hsa_circ_0045602–hsa-miR-4538–WNT5A and hsa_circ_0018004–hsa-miR-199a-5p–KL.

### Enrichment Analysis of Immune-Related mRNAs in the ceRNA Regulatory Network

**Figure 4:**
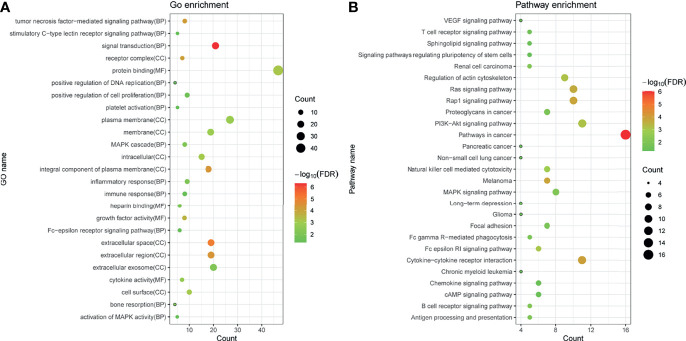
GO **(A)** and KEGG **(B)** enrichment analyses of immune-related mRNAs in the regulatory ceRNA network in GC. BP, biological process; CC, cytological component; MF, molecular function.

Fifty-eight immune-related mRNAs in the regulatory ceRNA network were used for functional enrichment analysis. GO analysis showed that signal transduction, extracellular space, and growth factor activity were the most significantly enriched biological process, cytological component, and molecular function, respectively (**Figure 4**). According to the KEGG analysis, Rap1 and Ras signaling pathways [involved NRAS proto-oncogene, GTPase (NRAS), and fibroblast growth factor 10 (FGF10)], Fc gamma R-mediated phagocytosis and cAMP signaling pathway [involved Rac family small GTPase 1 (RAC1)], proteoglycans in cancer [involved MET proto-oncogene, receptor tyrosine kinase (MET)], T-cell receptor signaling pathway [involved mitogen-activated protein kinase kinase 1 (MAP2K1)], and chemokine signaling pathway [involved LYN proto-oncogene, Src family tyrosine kinase (LYN)] were significantly enriched signaling pathways (**Figure 4** and **Table 5**).

**Table 5:** KEGG enrichment analysis of immune-related mRNAs in the ceRNA regulatory network in GC.

Term	Count	*p*-value	FDR	mRNAs
hsa05200:Pathways in cancer	16	8.65E−09	9.34E−07	MAP2K1, PRKCB, F2R, WNT5A, TGFBR1, PGF, NRAS, FGF14, TRAF3, CDK4, AGTR1, RAC1, FGF13, BID, MET, FGF10
hsa04060:Cytokine–cytokine receptor interaction	11	2.17E−06	1.17E−04	GHR, CXCL9, TNFRSF12A, TNFSF15, TNFRSF9, LIF, TNFSF11, TNFRSF10B, LIFR, IL1RAP, TGFBR1
hsa05218:Melanoma	7	5.05E−06	1.47E−04	MAP2K1, NRAS, FGF14, CDK4, FGF13, MET, FGF10
hsa04015:Rap1 signaling pathway	10	5.43E−06	1.47E−04	MAP2K1, NRAS, FGF14, PRKCB, F2R, RAC1, FGF13, MET, PGF, FGF10
hsa04014:Ras signaling pathway	10	9.89E−06	2.14E−04	MAP2K1, NRAS, FGF14, PRKCB, RAC1, FGF13, PAK3, MET, PGF, FGF10
hsa04810:Regulation of actin cytoskeleton	9	4.54E−05	7.33E−04	MAP2K1, NRAS, FGF14, F2R, RAC1, FGF13, PAK3, VAV2, FGF10
hsa04151:PI3K–Akt signaling pathway	11	4.75E−05	7.33E−04	GHR, MAP2K1, NRAS, FGF14, CDK4, F2R, RAC1, FGF13, MET, PGF, FGF10
hsa04664:Fc epsilon RI signaling pathway	6	6.55E−05	8.85E−04	LYN, MAP2K1, NRAS, PRKCB, RAC1, VAV2
hsa04650:Natural killer cell mediated cytotoxicity	7	1.12E−04	0.001343	MAP2K1, NRAS, PRKCB, TNFRSF10B, RAC1, BID, VAV2
hsa05211:Renal cell carcinoma	5	7.86E−04	0.008494	MAP2K1, NRAS, RAC1, PAK3, MET
hsa04662:B-cell receptor signaling pathway	5	9.30E−04	0.009003	LYN, MAP2K1, NRAS, RAC1, VAV2
hsa04010:MAPK signaling pathway	8	0.001	0.009003	MAP2K1, NRAS, FGF14, PRKCB, RAC1, FGF13, TGFBR1, FGF10
hsa04612:Antigen processing and presentation	5	0.001337	0.011104	PSME3, TAP2, TAP1, CALR, CTSB
hsa05205:Proteoglycans in cancer	7	0.001573	0.012138	MAP2K1, NRAS, PRKCB, WNT5A, PLAUR, RAC1, MET
hsa04510:Focal adhesion	7	0.00183	0.013074	MAP2K1, PRKCB, RAC1, PAK3, MET, PGF, VAV2
hsa04666:Fc gamma R-mediated phagocytosis	5	0.001937	0.013074	LYN, MAP2K1, PRKCB, RAC1, VAV2
hsa04660:T-cell receptor signaling pathway	5	0.003655	0.023221	MAP2K1, NRAS, CDK4, PAK3, VAV2
hsa05223:Non-small cell lung cancer	4	0.005345	0.032068	MAP2K1, NRAS, PRKCB, CDK4
hsa04062:Chemokine signaling pathway	6	0.006326	0.034272	LYN, MAP2K1, CXCL9, NRAS, RAC1, VAV2
hsa04730:Long-term depression	4	0.006482	0.034272	LYN, MAP2K1, NRAS, PRKCB
hsa04370:VEGF signaling pathway	4	0.006787	0.034272	MAP2K1, NRAS, PRKCB, RAC1
hsa04071:Sphingolipid signaling pathway	5	0.006981	0.034272	MAP2K1, NRAS, PRKCB, RAC1, BID
hsa05214:Glioma	4	0.008093	0.035418	MAP2K1, NRAS, PRKCB, CDK4
hsa05212:Pancreatic cancer	4	0.008093	0.035418	MAP2K1, CDK4, RAC1, TGFBR1
hsa04024:cAMP signaling pathway	6	0.008199	0.035418	MAP2K1, FSHB, F2R, RAC1, ADRB2, VAV2
hsa05220:Chronic myeloid leukemia	4	0.010713	0.044498	MAP2K1, NRAS, CDK4, TGFBR1
hsa04550:Signaling pathways regulating pluripotency of stem cells	5	0.01188	0.047521	MAP2K1, NRAS, WNT5A, LIF, LIFR

FDR, false discovery rate.

### PPI Network of Immune-Related mRNAs in the ceRNA Regulatory Network

**Figure 5:**
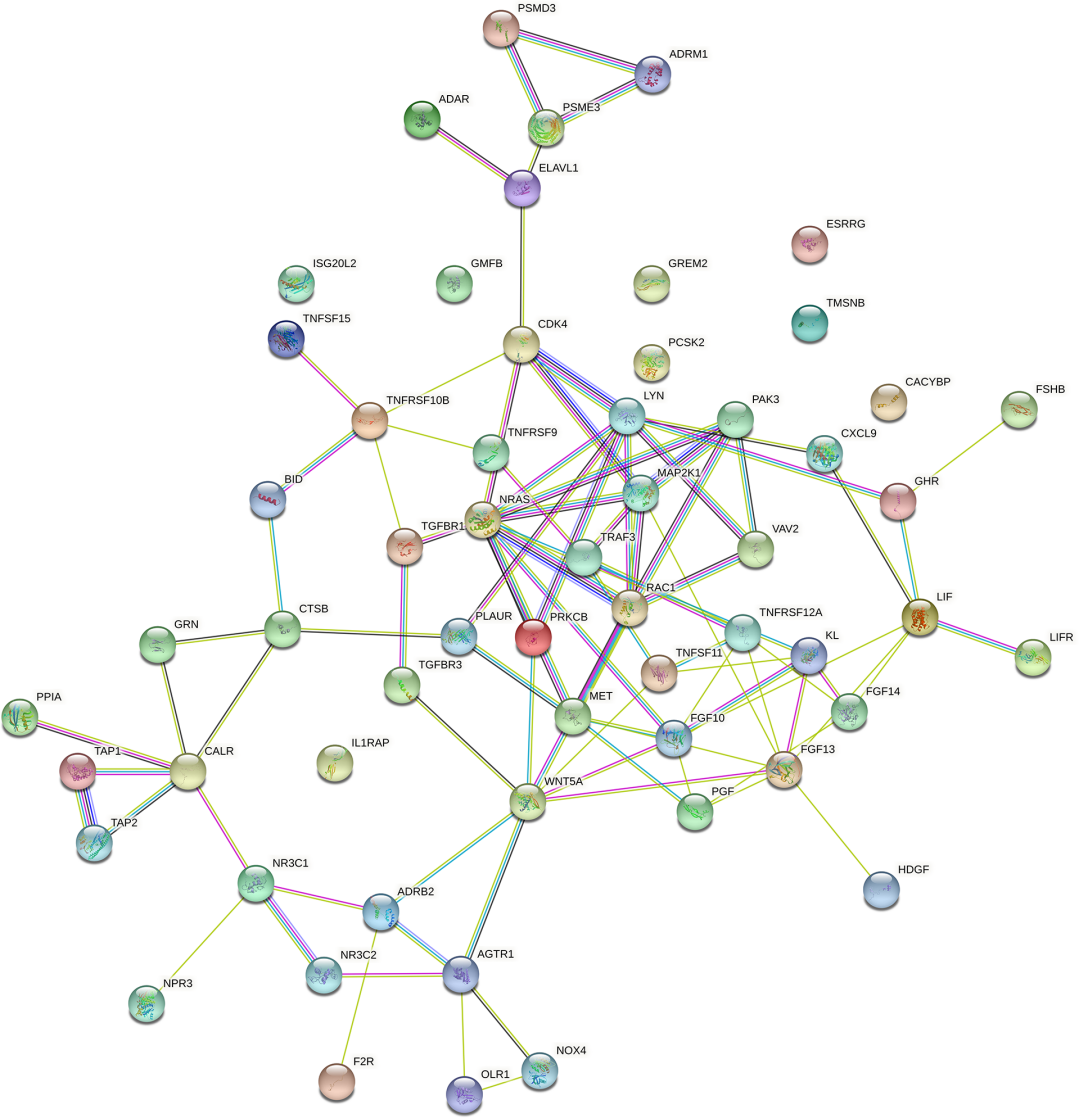
The PPI network of immune-related mRNAs in the regulatory ceRNA network in GC.

**Figure 6:**
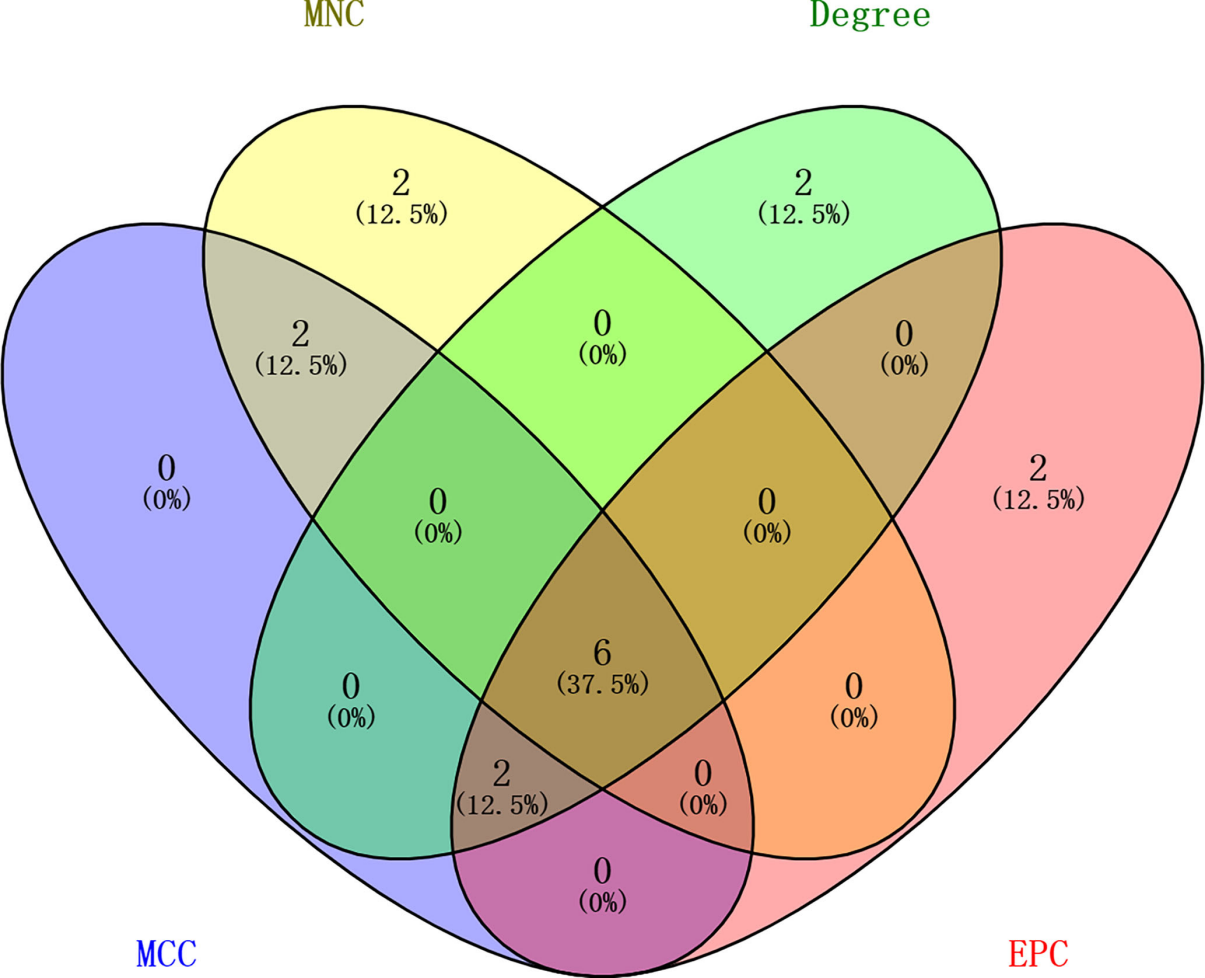
Identification of the six core immune-related mRNAs in the PPI network *via* four algorithms in GC.

**Figure 7:**
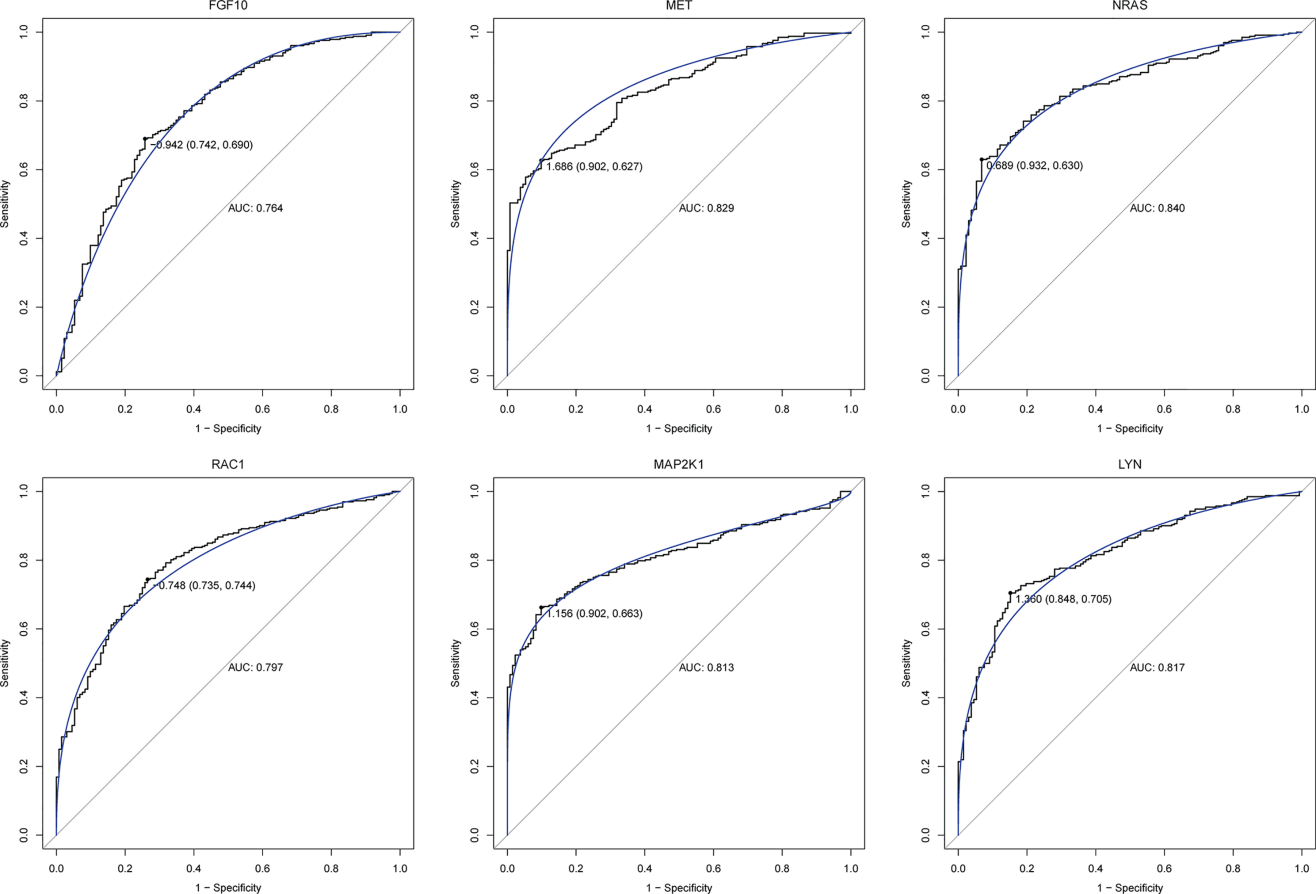
ROC analysis of six core immune-related mRNAs in the PPI network in GC.

**Figure 8:**
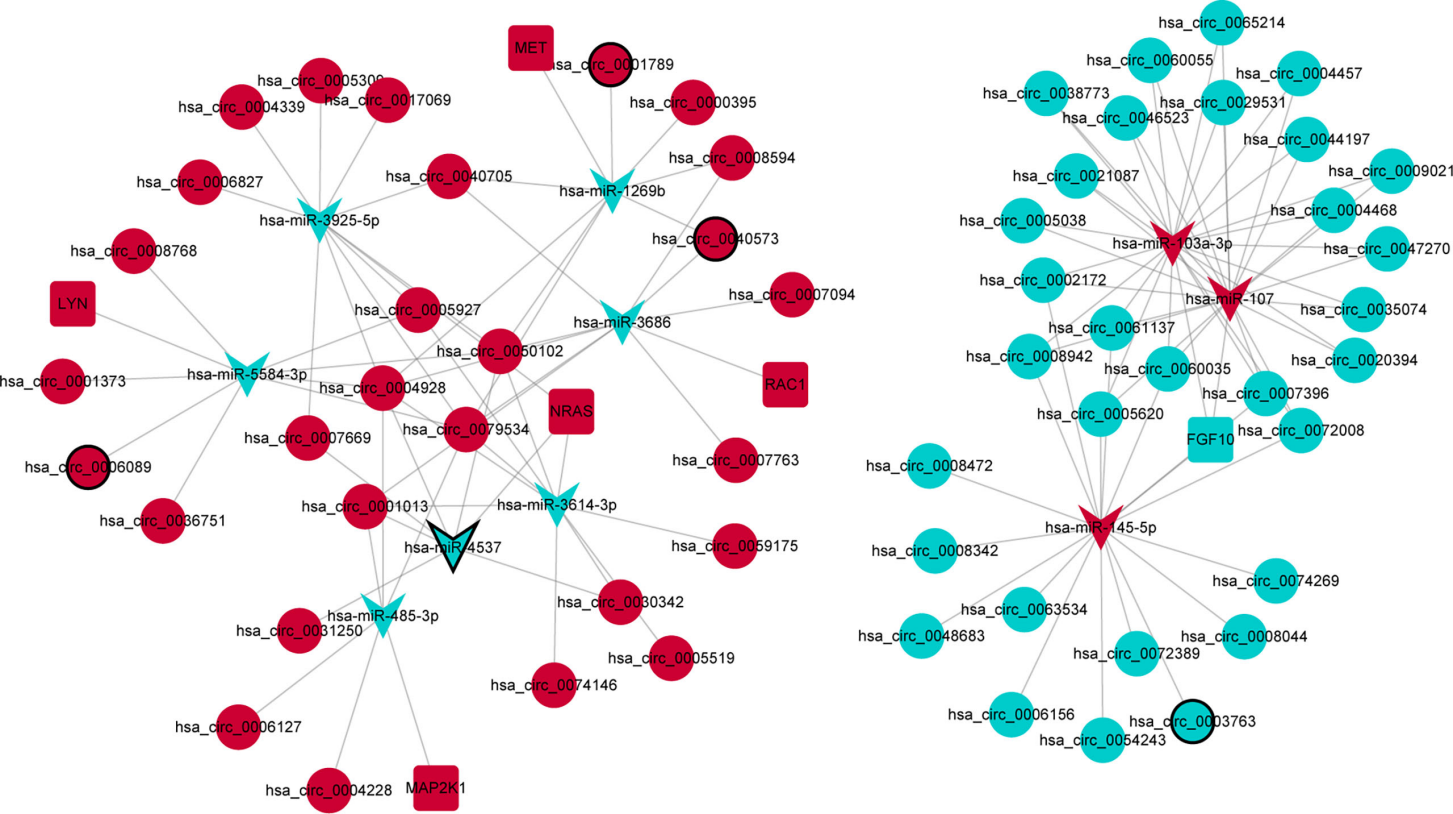
The ceRNA subnetwork based on six core immune-related mRNAs in the PPI network in GC. Circle, V shape, and rectangle represent differentially expressed circRNA, miRNA, and mRNA, respectively. Red and green represent upregulation and downregulation, respectively. The black border represents the top 10 up/downregulated circRNAs/miRNAs/mRNAs.

**Figure 9:**
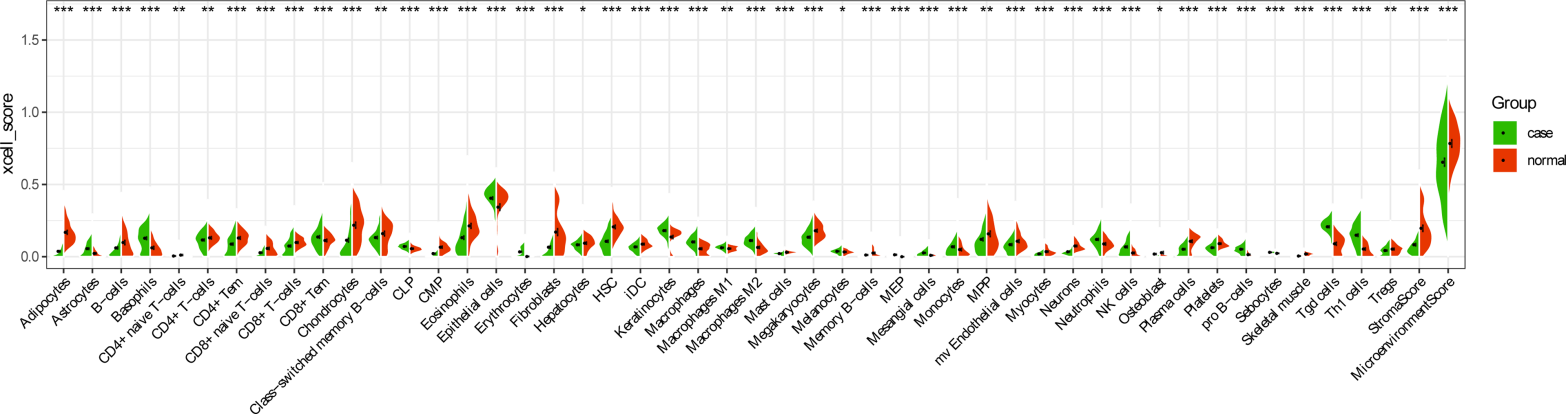
Distribution of immune cell types in GC. *p < 0.05; **p < 0.01; ***p < 0.001.

**Figure 10:**
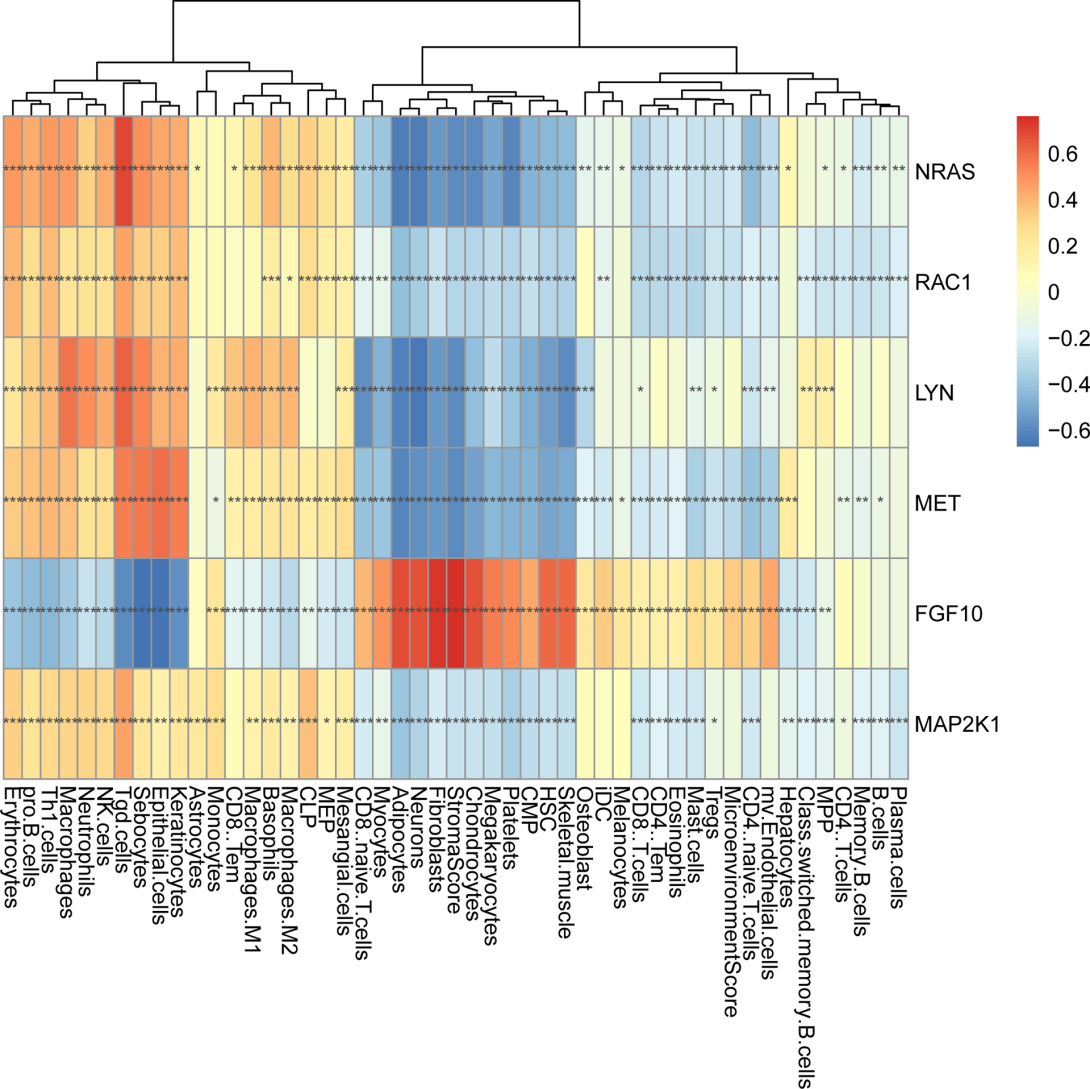
The correlation analysis between six core immune-related mRNAs and immune cells in GC. *p < 0.05; **p < 0.01; ***p < 0.001.

In order to further explore the interaction between immune-related mRNAs in the regulatory ceRNA network, the PPI network was established (**Figure 5**). Six core immune-related mRNAs were identified by four algorithms, namely, FGF10, MET, NRAS, RAC1, MAP2K1, and LYN (**Figure 6**). It is worth mentioning that FGF10 (AUC = 0.764), MET (AUC = 0.829), NRAS (AUC = 0.840), RAC1 (AUC = 0.797), MAP2K1 (AUC = 0.813), and LYN (AUC = 0.817) had a potential diagnostic value for GC (**Figure 7**). It was indicated that these mRNAs had a diagnostic potential for GC. In addition, the ceRNA subnetwork (involving 59 circRNAs and 10 miRNAs) based on the above six mRNAs was constructed (**Figure 8**). The ceRNA subnetwork contains 75 nodes and 124 edges. Some ceRNA relationship pairs were identified, such as hsa_circ_0050102–hsa-miR-4537–NRAS, hsa_circ_0001013–hsa-miR-485-3p–MAP2K1, hsa_circ_0003763–hsa-miR-145-5p–FGF10, hsa_circ_0001789–hsa-miR-1269b–MET, hsa_circ_0040573–hsa-miR-3686–RAC1, and hsa_circ_0006089–hsa-miR-5584-3p–LYN. In addition, xCell was used to calculate the distribution of immune cells in each sample based on the ssGSEA method. The types of immune cells were different in the GC group from those in the normal control group. For example, Tgd cells and adipocytes were the most upregulated and downregulated types of immune cells in GC (**Figure 9**). The Pearson correlation coefficient method was used to calculate the correlation between above six mRNAs of differential immune cells (**Figure 10**). The result showed that NRAS, RAC1, and MAP2K1 were positively correlated with Tgd cells. FGF10 was positively correlated with StromaScore. LYN and MET were negatively correlated with neurons and adipocytes, respectively.

### Expression Validation of Differentially Expressed circRNAs and Immune-Related mRNAs

**Figure 11:**
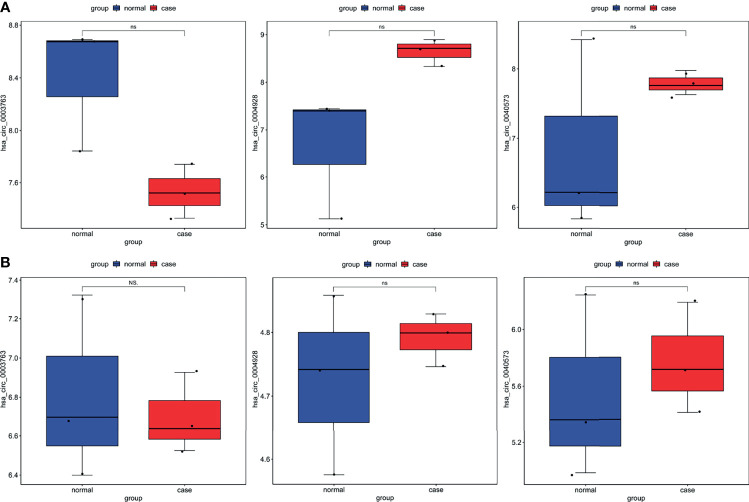
Expression validation of hsa_circ_0003763, hsa_circ_0004928, and hsa_circ_0040573 in the GSE93541 dataset **(A)** and GSE141977 dataset **(B)**. Ns, no significant difference.

**Figure 12:**
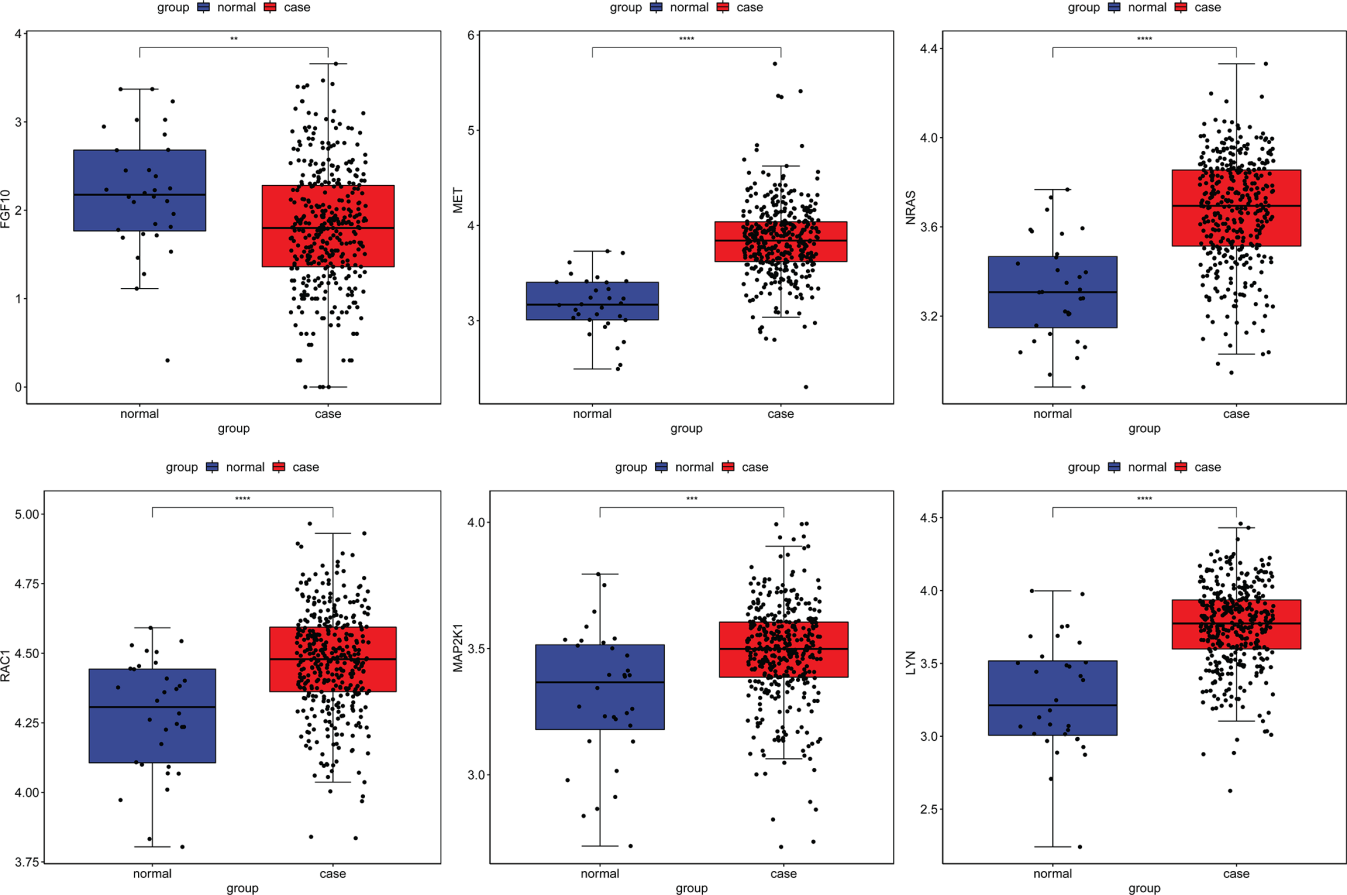
Expression validation of FGF10, MET, NRAS, RAC1, MAP2K1, and LYN in the TCGA dataset. **p < 0.01; ***p < 0.001; ****p < 0.0001.

To further validate the expression of identified circRNAs and immune-related mRNAs, electronic validation was performed. The GSE93541 dataset (**Figure 11A**) and the GSE141977 dataset (**Figure 11B**) were used for expression validation of hsa_circ_0003763, hsa_circ_0004928, and hsa_circ_0040573. The result showed that hsa_circ_0003763 was downregulated and hsa_circ_0004928 and hsa_circ_0040573 were upregulated in GC. Although there was no significant difference (may be caused by the small sample size), the expression trend was consistent with the integrated analysis results. In addition, the TCGA dataset was used to validate the expression of FGF10, MET, NRAS, RAC1, MAP2K1, and LYN (**Figure 12**). The result showed that FGF10 was significantly downregulated. MET, NRAS, RAC1, MAP2K1, and LYN were remarkably upregulated in GC, which is in line with the integrated analysis results.

**Figure 13:**
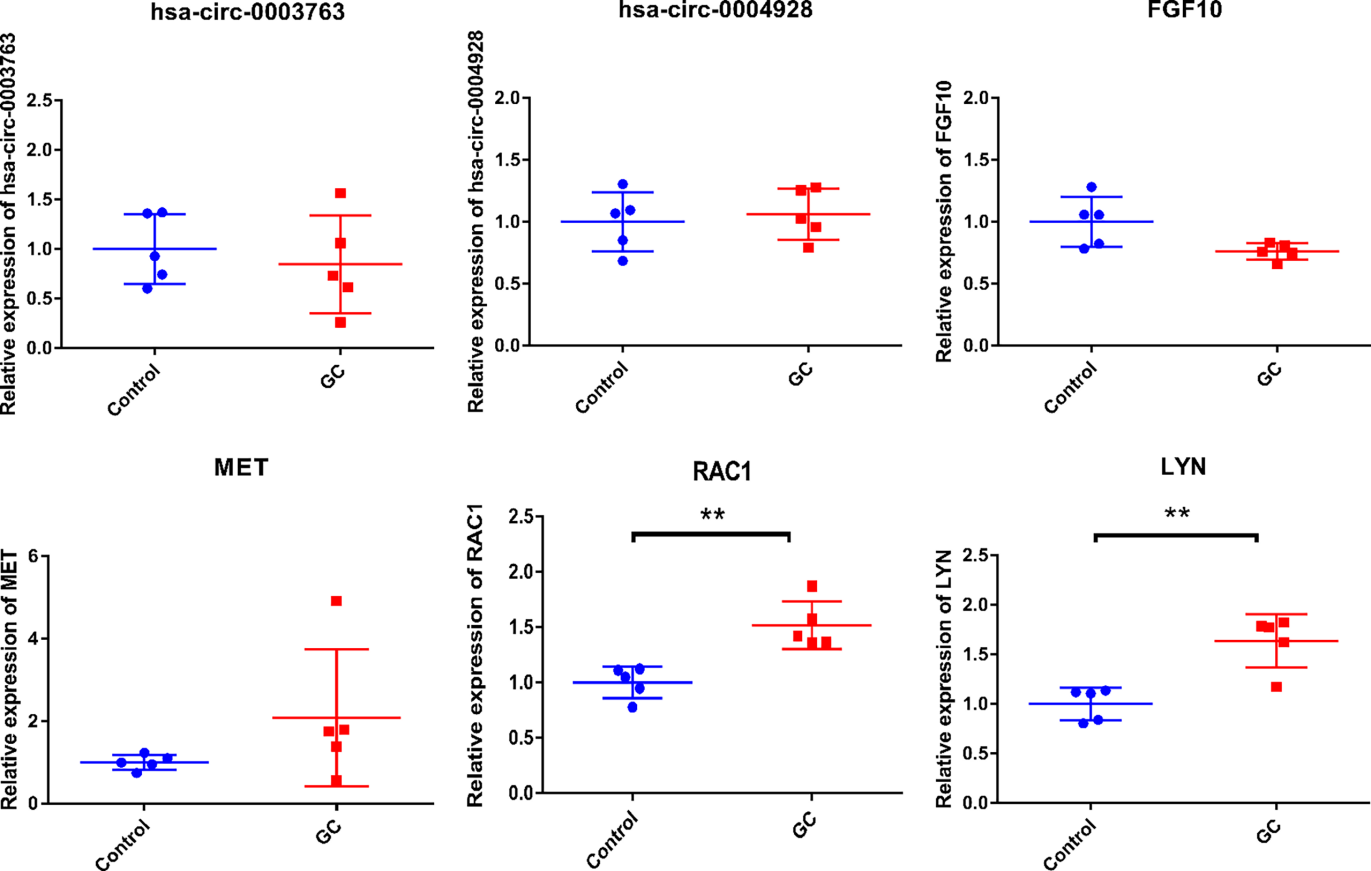
*In-vitro* validation of hsa_circ_0003763, hsa_circ_0004928, FGF10, MET, RAC1, and LYN. **p-value < 0.01.

To validate the expression of two circRNAs (hsa_circ_0003763 and hsa_circ_0004928) and four immune-related mRNAs (FGF10, MET, RAC1, and LYN), blood samples from five GC patients and five normal controls were collected for qRT-PCR (**Figure 13**). The clinical information of these individuals is listed in **Table 6**. The qRT-PCR result showed that RAC1 and LYN were significantly upregulated in GC, which was consistent with the integrated analysis results, while the expression trends of hsa_circ_0004928, MET, hsa_circ_0003763, and FGF10 were the same with the integrated analysis results without statistical significance.

**Table 6:** Clinical information of individuals in the *in-vitro* experiment.

Group	Age (years)	Gender	Height (cm)	Weight (kg)	Pathological grading	Clinical staging			Differentiated degree	Metastasis	Metastatic site	Smoking history	Drinking history
					Stage I, II, III, IV	T	N	M	(G1, 2, 3, 4)				
Control 1	63	Female	158	52	NA	NA	NA	NA	NA	NA	NA	No	No
Control 2	45	Female	156	54.5	NA	NA	NA	NA	NA	NA	NA	No	No
Control 3	66	Female	161	57	NA	NA	NA	NA	NA	NA	NA	No	No
Control 4	39	Female	159	59	NA	NA	NA	NA	NA	NA	NA	No	No
Control 5	59	Female	153	57	NA	NA	NA	NA	NA	NA	NA	No	No
GC 1	66	Male	170	73.2	IA	pT1a	0	0	G3	No	No	No	No
GC 2	58	Male	173	49	IIIB	pT4a	3a	0	G1	No	No	No	No
GC 3	70	Male	160	48	cIVB	cT4a	x	1	G3	Yes	Cervical lymph node	No	No
GC 4	58	Male	168	63.9	IV	cTx	x	1	Gx	Yes	Liver	Yes	Yes
GC 5	54	Female	158	56.6	IV	cT4	x	1	Gx	Yes	Abdominal cavity, left adnexa, and ascites	Yes	No

NA, not applicable.

## Discussion

Up to now, no articles about hsa_circ_0050102 have been reported in any diseases. hsa_circ_0003763 was associated with pancreatic ductal adenocarcinoma (22). hsa_circ_0003763 was upregulated in hepatocellular carcinoma tissues and facilitated the invasion of hepatocellular carcinoma cells (23). hsa-miR-4537 was associated with vitreoretinal lymphoma (24). hsa-miR-145-5p played key roles in GC epithelial cells and affected cell invasion and migration of GC cells (25). In GC cells, hsa-miR-145-5p has been verified as the direct target of has_circ_0000376 (26). It has been demonstrated that hsa_circ_DLST can act as the sponge of has-miR-502-5p to regulate the NRAS/MAP kinase/ERK kinase 1/2 signaling pathways in GC cells (27). FGF10, a secreted factor, stimulated the proliferation of lung cancer cells (28). In GC, FGF10 was involved in several signaling pathways (29). Tgd cells were related to the prognosis of patients with stomach adenocarcinoma (30). In this study, we found regulatory pairs of hsa_circ_0050102–hsa-miR-4537–NRAS–Tgd cells and hsa_circ_0003763–hsa-miR-145-5p–FGF10–StromaScore in GC, among which, NRAS and FGF10 had potential diagnostic value for patients. In addition, NRAS and FGF10 were involved in both Rap1 and Ras signaling pathways in GC. Rap1 played important roles in the metastasis and invasion of various tumor cells by regulating cytoskeleton remodeling. In GC, some differentially expressed angiogenesis-related genes were associated with the Rap1 signaling pathway (31). Genes involved in the Ras signaling pathway were found in approximately 40% of GC patients (32). It has been suggested that regulatory networks of hsa_circ_0050102–hsa-miR-4537–NRAS–Tgd cells and hsa_circ_0003763–hsa-miR-145-5p–FGF10–StromaScore may play important roles in the proliferation, migration, and invasion of GC by involving signaling pathways of Rap1 and Ras.

hsa_circ_0040573 was found in human umbilical vein endothelial cell line (33). It was shown that RAC1 was an essential effector of GC malignant transformation and metastasis (34, 35). In gastric cancer tissues, the expression of RAC1 was increased, which was significantly related to TNM stage (36). Herein, we found the regulatory pairs of hsa_circ_0040573–hsa-miR-3686–RAC1–Tgd cells in GC. It was noted that RAC1 had potential diagnostic value for patients. Moreover, RAC1 was associated with Fc gamma R-mediated phagocytosis and cAMP signaling pathway. Fc gamma R-mediated phagocytosis was found in plasma exosomes from GC patients (37). It has been demonstrated that there was a significant positive correlation between nuclear factor kappa B (NF-κB) and cAMP-regulated phosphoprotein expression level in GC tissues (38). Thus, it can be seen that hsa_circ_0040573, RAC1, and Tgd cells played a crucial role in malignant transformation, metastasis, and TNM stage of GC.

The expression of hsa_circ_0001789 was found in GC (39). hsa-miR-1269b was downregulated in basal cell carcinoma and GC (40, 41). MET can be internalized by macrophages to educate them toward a protumorigenic phenotype (42). In addition, the upregulated paracrine hepatocyte growth factor can bind the c-MET receptor on the migrated GC cells to facilitate the proliferation of metastatic GC cells (43). In fat tissues, adipocytes regulated cancer development by their effects on the microenvironment. A previous report preliminarily showed that omental adipocytes could promote GC cell invasiveness (44). Herein, we found a regulatory pair of hsa_circ_0001789–hsa-miR-1269b–MET–adipocytes in GC. MET had a potential diagnostic value for patients. Furthermore, MET was involved in proteoglycans in cancer in GC. In gastric epithelia, the basal surface of cells was surrounded by the basement membrane, which was mainly composed of glycoproteins (45–47). The proteoglycan signaling pathway has been found in GC (48). It was indicated that the regulatory pair of hsa_circ_0001789–hsa-miR-1269b–MET–adipocytes played roles in cell proliferation and invasiveness of GC.

hsa_circ_0001013, upregulated in GC, regulated the expression of fibrillin 1 through competing with miRNA response elements of hsa-miRNA-182-5p, which led to metastasis in GC (49). hsa-miR-485-3p, a key regulator of gastrointestinal stromal tumors, served as a molecular biomarker and potential therapeutic target for this malignant disease (50). The role of hsa-miR-485-3p has been found in GC cells (51). MAP2K1 was involved in the PD-L1 pathway in GC (52). In our study, we found the regulatory network of hsa_circ_0001013–hsa-miR-485-3p–MAP2K1–Tgd cells in GC, among which, MAP2K1 had a potential diagnostic value for GC patients. In addition, MAP2K1 was enriched in T-cell receptor signaling pathway. Our result indicated that hsa_circ_0001013, hsa-miR-485-3p, MAP2K1, and Tgd cells may be associated with tumor metastasis in the development of GC.

hsa-miR-5584-3p played important roles in tongue squamous cell carcinoma by interacting with hsa_circ_087212 (53). LYN was significantly downregulated in gastric gastrointestinal stromal tumors with high-grade malignancy (54). In this study, we found the relationship between hsa_circ_0006089, hsa-miR-5584-3p, and LYN. Moreover, LYN had a potential diagnostic value and was negatively correlated with neurons. In addition, LYN was involved in chemokine signaling pathway. It was suggested that chemokines and their specific receptors can play an important role in GC progression *via* promotion of angiogenesis, invasion, survival, and metastasis (55). This indicated that the regulatory network of hsa_circ_0006089–hsa-miR-5584-3p–LYN–neurons played important roles in angiogenesis, invasion, survival, and metastasis of GC.

Besides the above circRNA–miRNA–immune-related mRNA regulatory network, some other circRNA–miRNA–mRNA regulatory networks were also found in GC, such as hsa_circ_0045602–hsa-miR-4538–WNT5A and hsa_circ_0018004–hsa-miR-199a-5p–KL. hsa_circ_0045602 was upregulated in GC tumor tissues (56). The expression of hsa-miR-4538 was decreased in acute myeloid leukemia (57). hsa-miR-199a-5p level was significantly increased in GC tissues. Moreover, a higher miR-199a-5p expression level of hsa-miR-199a-5p promoted the invasion of GC cells and was related to increased likelihood of lymph node metastasis (58). Thus, it can be seen that these molecules may be involved in tumor cell invasion and metastasis of GC.

In conclusion, our study found several ceRNA (circRNA–miRNA–immune-related mRNA) regulatory networks including hsa_circ_0050102–hsa-miR-4537–NRAS–Tgd cells, hsa_circ_0001013–hsa-miR-485-3p–MAP2K1–Tgd cells, hsa_circ_0003763–hsa-miR-145-5p–FGF10–StromaScore, hsa_circ_0001789–hsa-miR-1269b–MET–adipocytes, hsa_circ_0040573–hsa-miR-3686–RAC1–Tgd cells, and hsa_circ_0006089–hsa-miR-5584-3p–LYN–neurons in GC, among which FGF10, MET, NRAS, RAC1, MAP2K1, and LYN had a potential diagnostic value for GC patients. In addition, some signaling pathways were identified, such as Rap1 and Ras signaling pathways (involved NRAS and FGF10), Fc gamma R-mediated phagocytosis and cAMP signaling pathway (involved RAC1), proteoglycans in cancer (involved MET), T-cell receptor signaling pathway (involved MAP2K1), and chemokine signaling pathway (involved LYN). Our study may provide a novel field for understanding the molecular mechanisms of GC at the immunological levels. However, there are limitations to our study. Firstly, larger numbers of samples in qRT-PCR are needed. Secondly, an *in-vitro* gastric cell line experiment in cultures and tissues from healthy and gastric cancer patients is further needed to confirm the results of the *in-silico* analyses. Thirdly, the cell reporter assay for miRNA–circRNA–mRNA interaction is further needed.

## Data Availability Statement

The original contributions presented in the study are included in the article/supplementary material. Further inquiries can be directed to the corresponding author.

## Ethics Statement

All participating individuals provided informed consent with the approval of the Ethics Committee of Zhejiang Hospital (2021-164K). The patients/participants provided their written informed consent to participate in this study.

## Author Contributions

GZ was the major contributor in the subject design. GZ contributed administrative support. PL provided the materials and samples and collected and sorted the data. ZW and GZ contributed to the data analysis and interpretation. All authors read and approved the final manuscript.

## Conflict of Interest

The authors declare that the research was conducted in the absence of any commercial or financial relationships that could be construed as a potential conflict of interest.

## Publisher’s Note

All claims expressed in this article are solely those of the authors and do not necessarily represent those of their affiliated organizations, or those of the publisher, the editors and the reviewers. Any product that may be evaluated in this article, or claim that may be made by its manufacturer, is not guaranteed or endorsed by the publisher.

## References

[1] Torre LA Bray F Siegel RL Ferlay J Lortet-Tieulent J Jemal A. Global Cancer Statistics, 2012. CA: Cancer J Clin (2015) 65(2):87–108. doi: 10.3322/caac.21262 25651787

[2] Sung H Ferlay J Siegel RL. Global Cancer Statistics 2020: GLOBOCAN Estimates of Incidence and Mortality Worldwide for 36 Cancers in 185 Countries. CA Cancer J Clin (2021) 71: (3):209–49. doi: 10.3322/caac.21660 33538338

[3] Ferlay J Colombet M Soerjomataram I Parkin DM. Cancer Statistics for the Year 2020: An Overview. Int J Cancer (2021). doi: 10.1002/ijc.33588 33818764

[4] Parkin DM. The Global Health Burden of Infection-Associated Cancers in the Year 2002. Int J Cancer (2006) 118(12):3030–44. doi: 10.1002/ijc.21731 16404738

[5] Al-Mahrouqi H Parkin L Sharples K. Incidence of Stomach Cancer in Oman and the Other Gulf Cooperation Council Countries. Oman Med J (2011) 26(4):258–62. doi: 10.5001/omj.2011.62 PMC3191710 22043430

[6] IARC Working Group on the Evaluation of Carcinogenic Risks to Humans. Personal Habits and Indoor Combustions. Volume 100 E. A Review of Human Carcinogens. IARC Monographs on the Evaluation of Carcinogenic Risks to Humans. IARC Monogr Eval Carcinog Risks Hum (2012) 100: (Pt E):1–538. PMC4781577 23193840

[7] Plummer M Franceschi S Vignat J Forman D de Martel C. Global Burden of Gastric Cancer Attributable to Helicobacter Pylori. Int J Cancer (2015) 136(2):487–90. doi: 10.1002/ijc.28999 24889903

[8] Moss SF. The Clinical Evidence Linking Helicobacter Pylori to Gastric Cancer. Cell Mol Gastroenterol Hepatol (2017) 3(2):183–91. doi: 10.1016/j.jcmgh.2016.12.001 PMC5331857 28275685

[9] Nagini S. Carcinoma of the Stomach: A Review of Epidemiology, Pathogenesis, Molecular Genetics and Chemoprevention. World J Gastrointest Oncol (2012) 4(7):156–69. doi: 10.4251/wjgo.v4.i7.156 PMC3406280 22844547

[10] Van Cutsem E Sagaert X Topal B Haustermans K Prenen H. Gastric Cancer. Lancet (London England) (2016) 388(10060):2654–64. doi: 10.1016/S0140-6736(16)30354-3 27156933

[11] Petkovic S Müller S. RNA Circularization Strategies *In Vivo* and *In Vitro* . Nucleic Acids Res (2015) 43(4):2454–65. doi: 10.1093/nar/gkv045 PMC4344496 25662225

[12] Pereira AL Magalhães L Pantoja RP Araújo G Ribeiro-Dos-Santos Â Vidal AF. The Biological Role of Sponge Circular RNAs in Gastric Cancer: Main Players or Coadjuvants? Cancers (Basel) (2020) 12 (7):1982. doi: 10.3390/cancers12071982 PMC7409348 32708088

[13] Zhang J Liu H Hou L Wang G Zhang R Huang Y. Circular RNA_LARP4 Inhibits Cell Proliferation and Invasion of Gastric Cancer by Sponging miR-424-5p and Regulating LATS1 Expression. Mol Cancer (2017) 16(1):151. doi: 10.1186/s12943-017-0719-3 28893265 PMC5594516

[14] Zhang X Wang S Wang H Cao J Huang X Chen Z. Circular RNA Circnrip1 Acts as a microRNA-149-5p Sponge to Promote Gastric Cancer Progression *via* the AKT1/mTOR Pathway. Mol Cancer (2019) 18(1):20. doi: 10.1186/s12943-018-0935-5 30717751 PMC6360801

[15] Zhu Z Rong Z Luo Z Yu Z Zhang J Qiu Z. Circular RNA Circnhsl1 Promotes Gastric Cancer Progression Through the miR-1306-3p/SIX1/vimentin Axis. Mol Cancer (2019) 18(1):126. doi: 10.1186/s12943-019-1054-7 31438963 PMC6704702

[16] Zhang L Song X Chen X Wang Q Zheng X Wu C. Circular RNA CircCACTIN Promotes Gastric Cancer Progression by Sponging MiR-331-3p and Regulating TGFBR1 Expression. Int J Biol Sci (2019) 15(5):1091–103. doi: 10.7150/ijbs.31533 PMC6535790 31182928

[17] Zhang Z Yu X. Circular RNA Circ_0026359 Enhances Cisplatin Resistance in Gastric Cancer *via* Targeting miR-1200/POLD4 Pathway. Biomed Res Int (2020) 2020:. doi: 10.1155/2020/5103272 PMC7443216 32855967

[18] Wang F Meng W Wang B Qiao L. Helicobacter Pylori-Induced Gastric Inflammation and Gastric Cancer. Cancer Lett (2014) 345(2):196–202. doi: 10.1016/j.canlet.2013.08.016 23981572

[19] Yolanda LV Sergio PD Hugo ES Isabel AF Rafael BZ Aldo TD. Gastric Cancer Progression Associated With Local Humoral Immune Responses. BMC Cancer (2015) 15:924. doi: 10.1186/s12885-015-1858-9 26589831 PMC4654873

[20] Tsujimoto H Ono S Ichikura T Matsumoto Y Yamamoto J Hase K. Roles of Inflammatory Cytokines in the Progression of Gastric Cancer: Friends or Foes? Gastric Cancer (2010) 13(4):212–21. doi: 10.1007/s10120-010-0568-x 21128056

[21] Aran D Hu Z Butte AJ. Xcell: Digitally Portraying the Tissue Cellular Heterogeneity Landscape. Genome Biol (2017) 18(1):220. doi: 10.1186/s13059-017-1349-1 29141660 PMC5688663

[22] Guo X Zhou Q Su D Luo Y Fu Z Huang L. Circular RNA circBFAR Promotes the Progression of Pancreatic Ductal Adenocarcinoma *via* the miR-34b-5p/MET/Akt Axis. Mol Cancer (2020) 19(1):83. doi: 10.1186/s12943-020-01196-4 32375768 PMC7201986

[23] Li K Cao J Zhang Z Chen K Ma T Yang W. Circular RNA Circgsk3b Promotes Cell Proliferation, Migration, and Invasion by Sponging miR-1265 and Regulating CAB39 Expression in Hepatocellular Carcinoma. Front Oncol (2020) 10:598256. doi: 10.3389/fonc.2020.598256 33262952 PMC7688052

[24] Minezaki T Usui Y Asakage M Takanashi M Shimizu H Nezu N. High-Throughput MicroRNA Profiling of Vitreoretinal Lymphoma: Vitreous and Serum MicroRNA Profiles Distinct From Uveitis. J Clin Med (2020) 9(6):1844. doi: 10.3390/jcm9061844 PMC7356511 32545709

[25] Zhou K Song B Wei M Fang J Xu Y. MiR-145-5p Suppresses the Proliferation, Migration and Invasion of Gastric Cancer Epithelial Cells *via* the ANGPT2/NOD_LIKE_RECEPTOR Axis. Cancer Cell Int (2020) 20:416. doi: 10.1186/s12935-020-01483-6 32874130 PMC7456024

[26] Ju C Zhou J Miao H Chen X Zhang Q. Bupivacaine Suppresses the Progression of Gastric Cancer Through Regulating Circ_0000376/miR-145-5p Axis. BMC Anesthesiol (2020) 20(1):275. doi: 10.1186/s12871-020-01179-4 33126850 PMC7597012

[27] Zhang J Hou L Liang R Chen X Zhang R Chen W. CircDLST Promotes the Tumorigenesis and Metastasis of Gastric Cancer by Sponging miR-502-5p and Activating the NRAS/MEK1/ERK1/2 Signaling. Mol Cancer (2019) 18(1):80. doi: 10.1186/s12943-019-1015-1 30953514 PMC6449953

[28] Suzuki T Yasuda H Funaishi K Arai D Ishioka K Ohgino K. Multiple Roles of Extracellular Fibroblast Growth Factors in Lung Cancer Cells. Int J Oncol (2015) 46(1):423–9. doi: 10.3892/ijo.2014.2718 25353145

[29] Carino A Graziosi L Marchianò S Biagioli M Marino E Sepe V. Analysis of Gastric Cancer Transcriptome Allows the Identification of Histotype Specific Molecular Signatures With Prognostic Potential. Front Oncol (2021) 11:663771. doi: 10.3389/fonc.2021.663771 34012923 PMC8126708

[30] Hong C Yang S Wang Q Zhang S Wu W Chen J. Epigenetic Age Acceleration of Stomach Adenocarcinoma Associated With Tumor Stemness Features, Immunoactivation, and Favorable Prognosis. Front Genet (2021) 12:563051. doi: 10.3389/fgene.2021.563051 33815458 PMC8012546

[31] Cai WY Dong ZN Fu XT Lin LY Wang L Ye GD. Identification of a Tumor Microenvironment-Relevant Gene Set-Based Prognostic Signature and Related Therapy Targets in Gastric Cancer. Theranostics (2020) 10(19):8633–47. doi: 10.7150/thno.47938 PMC7392024 32754268

[32] Hatakeyama M. Helicobacter Pylori CagA and Gastric Cancer: A Paradigm for Hit-and-Run Carcinogenesis. Cell Host Microbe (2014) 15(3):306–16. doi: 10.1016/j.chom.2014.02.008 24629337

[33] O’Leary VB Smida J Matjanovski M Brockhaus C Winkler K Moertl S. The circRNA Interactome-Innovative Hallmarks of the Intra- and Extracellular Radiation Response. Oncotarget (2017) 8(45):78397–409. doi: 10.18632/oncotarget.19228 PMC5667970 29108237

[34] Xue Y Bi F Zhang X Pan Y Liu N Zheng Y. Inhibition of Endothelial Cell Proliferation by Targeting Rac1 GTPase With Small Interference RNA in Tumor Cells. Biochem Biophys Res Commun (2004) 320(4):1309–15. doi: 10.1016/j.bbrc.2004.06.088 15303276

[35] Xue Y Bi F Zhang X Zhang S Pan Y Liu N. Role of Rac1 and Cdc42 in Hypoxia Induced P53 and Von Hippel-Lindau Suppression and HIF1alpha Activation. Int J Cancer (2006) 118(12):2965–72. doi: 10.1002/ijc.21763 16395716

[36] Li P Chen X Su L Li C Zhi Q Yu B. Epigenetic Silencing of miR-338-3p Contributes to Tumorigenicity in Gastric Cancer by Targeting SSX2IP. PloS One (2013) 8(6):e66782. doi: 10.1371/journal.pone.0066782 23826132 PMC3691322

[37] Rao M Zhu Y Qi L Hu F Gao P. Circular RNA Profiling in Plasma Exosomes From Patients With Gastric Cancer. Oncol Lett (2020) 20(3):2199–208. doi: 10.3892/ol.2020.11800 PMC7403632 32765789

[38] Zhu S Soutto M Chen Z Peng D Romero-Gallo J Krishna US. Helicobacter Pylori-Induced Cell Death Is Counteracted by NF-κb-Mediated Transcription of DARPP-32. Gut (2017) 66(5):761–2. doi: 10.1136/gutjnl-2016-312141 PMC5334457 27590997

[39] Xia Y Lv J Jiang T Li B Li Y He Z. CircFAM73A Promotes the Cancer Stem Cell-Like Properties of Gastric Cancer Through the miR-490-3p/HMGA2 Positive Feedback Loop and HNRNPK-Mediated β-Catenin Stabilization. J Exp Clin Cancer Res: CR (2021) 40(1):103. doi: 10.1186/s13046-021-01896-9 33731207 PMC7972245

[40] Wei HP Zhan S Zhu QA Chen ZJ Feng X Chen JY. Genome-Wide Expression Difference of MicroRNAs in Basal Cell Carcinoma. J Immunol Res (2021) 2021:7223500. doi: 10.1155/2021/7223500 34395634 PMC8357504

[41] Zhang C Zhang CD Ma MH Dai DQ. Three-microRNA Signature Identified by Bioinformatics Analysis Predicts Prognosis of Gastric Cancer Patients. World J Gastroenterol (2018) 24(11):1206–15. doi: 10.3748/wjg.v24.i11.1206 PMC5859223 29568201

[42] Che Y Geng B Xu Y Miao X Chen L Mu X. Helicobacter Pylori-Induced Exosomal MET Educates Tumour-Associated Macrophages to Promote Gastric Cancer Progression. J Cell Mol Med (2018) 22(11):5708–19. doi: 10.1111/jcmm.13847 PMC6201349 30160350

[43] Huang T Song C Zheng L Xia L Li Y Zhou Y. The Roles of Extracellular Vesicles in Gastric Cancer Development, Microenvironment, Anti-Cancer Drug Resistance, and Therapy. Mol Cancer (2019) 18(1):62. doi: 10.1186/s12943-019-0967-5 30925929 PMC6441168

[44] Xiang F Wu K Liu Y Shi L Wang D Li G. Omental Adipocytes Enhance the Invasiveness of Gastric Cancer Cells by Oleic Acid-Induced Activation of the PI3K-Akt Signaling Pathway. Int J Biochem Cell Biol (2017) 84:14–21. doi: 10.1016/j.biocel.2016.12.002 27956048

[45] Frantz C Stewart KM Weaver VM. The Extracellular Matrix at a Glance. J Cell Sci (2010) 123(Pt 24):4195–200. doi: 10.1242/jcs.023820 PMC2995612 21123617

[46] Egeblad M Nakasone ES Werb Z. Tumors as Organs: Complex Tissues That Interface With the Entire Organism. Dev Cell (2010) 18(6):884–901. doi: 10.1016/j.devcel.2010.05.012 20627072 PMC2905377

[47] Xu R Boudreau A Bissell MJ. Tissue Architecture and Function: Dynamic Reciprocity *via* Extra- and Intra-Cellular Matrices. Cancer Metastasis Rev (2009) 28(1-2):167–76. doi: 10.1007/s10555-008-9178-z PMC2720096 19160017

[48] Sandoval-Bórquez A Polakovicova I Carrasco-Véliz N Lobos-González L Riquelme I Carrasco-Avino G. MicroRNA-335-5p Is a Potential Suppressor of Metastasis and Invasion in Gastric Cancer. Clin Epigenet (2017) 9:114. doi: 10.1186/s13148-017-0413-8 PMC5645854 29075357

[49] Tian Y Xing Y Zhang Z. Bioinformatics Analysis of Key Genes and circRNA-miRNA-mRNA Regulatory Network in Gastric Cancer. Biomed Res Int (2020) 2020. doi: 10.1155/2020/2862701 PMC7463386 32908877

[50] Jia N Tong H Zhang Y Katayama H Wang Y Lu W. CeRNA Expression Profiling Identifies KIT-Related circRNA-miRNA-mRNA Networks in Gastrointestinal Stromal Tumour. Front Genet (2019) 10:825. doi: 10.3389/fgene.2019.00825 31552107 PMC6746987

[51] Sun HD Xu ZP Sun ZQ Zhu B Wang Q Zhou J. Down-Regulation of Circpvrl3 Promotes the Proliferation and Migration of Gastric Cancer Cells. Sci Rep (2018) 8(1):10111. doi: 10.1038/s41598-018-27837-9 29973643 PMC6031698

[52] Du Z Yan D Li Z Gu J Tian Y Cao J. Genes Involved in the PD-L1 Pathway Might Associate With Radiosensitivity of Patients With Gastric Cancer. J Oncol (2020) 2020:7314195. doi: 10.1155/2020/7314195 32963532 PMC7495224

[53] Wei T Ye P Yu GY Zhang ZY. Circular RNA Expression Profiling Identifies Specific Circular RNAs in Tongue Squamous Cell Carcinoma. Mol Med Rep (2020) 21(4):1727–38. doi: 10.3892/mmr.2020.10980 PMC7057816 32319610

[54] Shen C Yin Y Chen H Wang R Yin X Cai Z. Secreted Protein Acidic and Rich in Cysteine-Like 1 Suppresses Metastasis in Gastric Stromal Tumors. BMC Gastroenterol (2018) 18(1):105. doi: 10.1186/s12876-018-0833-8 29973149 PMC6030747

[55] Pawluczuk E Łukaszewicz-Zając M Mroczko B. The Role of Chemokines in the Development of Gastric Cancer - Diagnostic and Therapeutic Implications. Int J Mol Sci (2020) 21(22):8456. doi: 10.3390/ijms21228456 PMC7697532 33182840

[56] Zhang Y Wang M Zang X Mao Z Chen Y Mao F. CircHN1 Affects Cell Proliferation and Migration in Gastric Cancer. J Clin Lab Anal (2020) 34: (10):e23433. doi: 10.1002/jcla.23433 32608539 PMC7595908

[57] Ma QL Wang JH Yang M Wang HP Jin J. MiR-362-5p as a Novel Prognostic Predictor of Cytogenetically Normal Acute Myeloid Leukemia. J Trans Med (2018) 16(1):68. doi: 10.1186/s12967-018-1445-3 PMC5853092 29540187

[58] He XJ Ma YY Yu S Jiang XT Lu YD Tao L. Up-Regulated miR-199a-5p in Gastric Cancer Functions as an Oncogene and Targets Klotho. BMC Cancer (2014) 14:218. doi: 10.1186/1471-2407-14-218 24655788 PMC3994330

